# Machine learning and statistical classification of birdsong link vocal acoustic features with phylogeny

**DOI:** 10.1038/s41598-023-33825-5

**Published:** 2023-05-01

**Authors:** Moises Rivera, Jacob A. Edwards, Mark E. Hauber, Sarah M. N. Woolley

**Affiliations:** 1 Department of Psychology, Hunter College and the Graduate Center, City University of New York, New York, NY 10065 USA; 2 Mortimer B. Zuckerman Mind, Brain, and Behavior Institute, Columbia University, New York, NY 10027 USA; 3 Department of Psychology, Columbia University, New York, NY 10027 USA; 4 Department of Evolution, Ecology, and Behavior, School of Biological Sciences, University of Illinois at Urbana-Champaign, Urbana, IL 61801 USA; 5 Zuckerman Institute at Columbia University, Jerome L. Greene Science Center, 3227 Broadway, L3.028, New York, NY 10027 USA

**Keywords:** Evolution, Climate sciences, Neuroscience, Computational neuroscience, Sexual behaviour, Social behaviour, Biological techniques, Behavioural methods, Machine learning

## Abstract

Birdsong is a longstanding model system for studying evolution and biodiversity. Here, we collected and analyzed high quality song recordings from seven species in the family *Estrildidae*. We measured the acoustic features of syllables and then used dimensionality reduction and machine learning classifiers to identify features that accurately assigned syllables to species. Species differences were captured by the first 3 principal components, corresponding to basic frequency, power distribution, and spectrotemporal features. We then identified the measured features underlying classification accuracy. We found that fundamental frequency, mean frequency, spectral flatness, and syllable duration were the most informative features for species identification. Next, we tested whether specific acoustic features of species’ songs predicted phylogenetic distance. We found significant phylogenetic signal in syllable frequency features, but not in power distribution or spectrotemporal features. Results suggest that frequency features are more constrained by species’ genetics than are other features, and are the best signal features for identifying species from song recordings. The absence of phylogenetic signal in power distribution and spectrotemporal features suggests that these song features are labile, reflecting learning processes and individual recognition.

## Introduction

Acoustic communication signals are a focus for understanding species evolution^1–7^, mating strategies^8–12^ and physiological mechanisms of behavior^13–16^. Many vertebrate species use vocalizations to convey species, sex, location, and behavioral state to receivers such as potential mates, competitors and family members^3,8,9,13,17–20^. Understanding the features of vocal signals that communicate specific social information requires a comparative analysis of species with spectrotemporal diversity and known relatedness in order to quantify how differences in the former may change across species based on the latter. In imitative vocal learners, such as songbirds, signal acoustics result from both genetic and experiential factors. Birdsong is a robust model behavior for studying mechanisms of plastic social communication; in turn, the evolution of song features is shaped by processes of cultural^8,21^, sexual^22^, and natural selection^9,23^.

Researchers have previously detected phylogenetic signal in the basic frequency features of the songs of *Regulus* birds, suggesting that differences in these features are closely related to differences in the phylogenetic relationships in these songbirds^24^. Frequency bandwidth and modulation of song syllables, however, did not carry a phylogenetic signal, suggesting that these features are shaped by learning processes^24^. Changes in song frequency features, on the other hand, may be constrained by pleiotropic or polygenic traits that affect vocal production organs (e.g., body size affecting fundamental and dominant frequencies through syrinx size)^25^ and other morphological features implicated in sound modification (e.g., the beak)^26^. These constraints would impart evolutionary rates on song frequency changes that correlate with time and reflect phylogenetic relationships, as opposed to faster rates of change typically found in song features that shift under processes of cultural transmission, including learning^27^.

**Figure 1:**
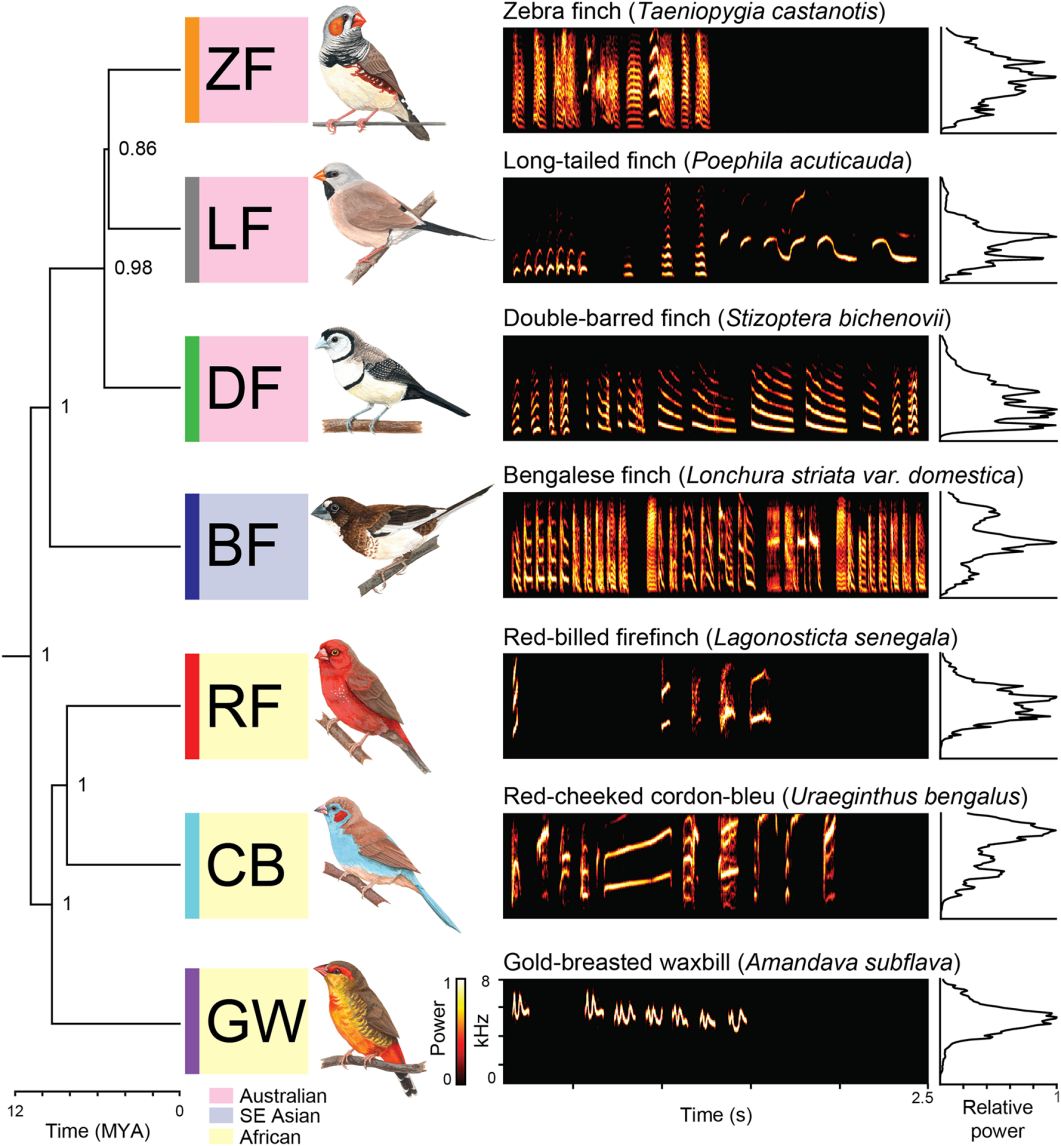
Phylogeny, sample spectrograms of song, and frequency power spectra of each species’ song in the 7 studied species. Left: Prior probabilities in the phylogenetic tree are labelled at each node. Center: Each spectrogram shows the spectral and temporal features of one bird’s song from each species, and the acoustic diversity of song across species. Right: Each frequency power spectrum to the right of a spectrogram shows the average distribution of power across acoustic frequencies for each species.

**Figure 2:**
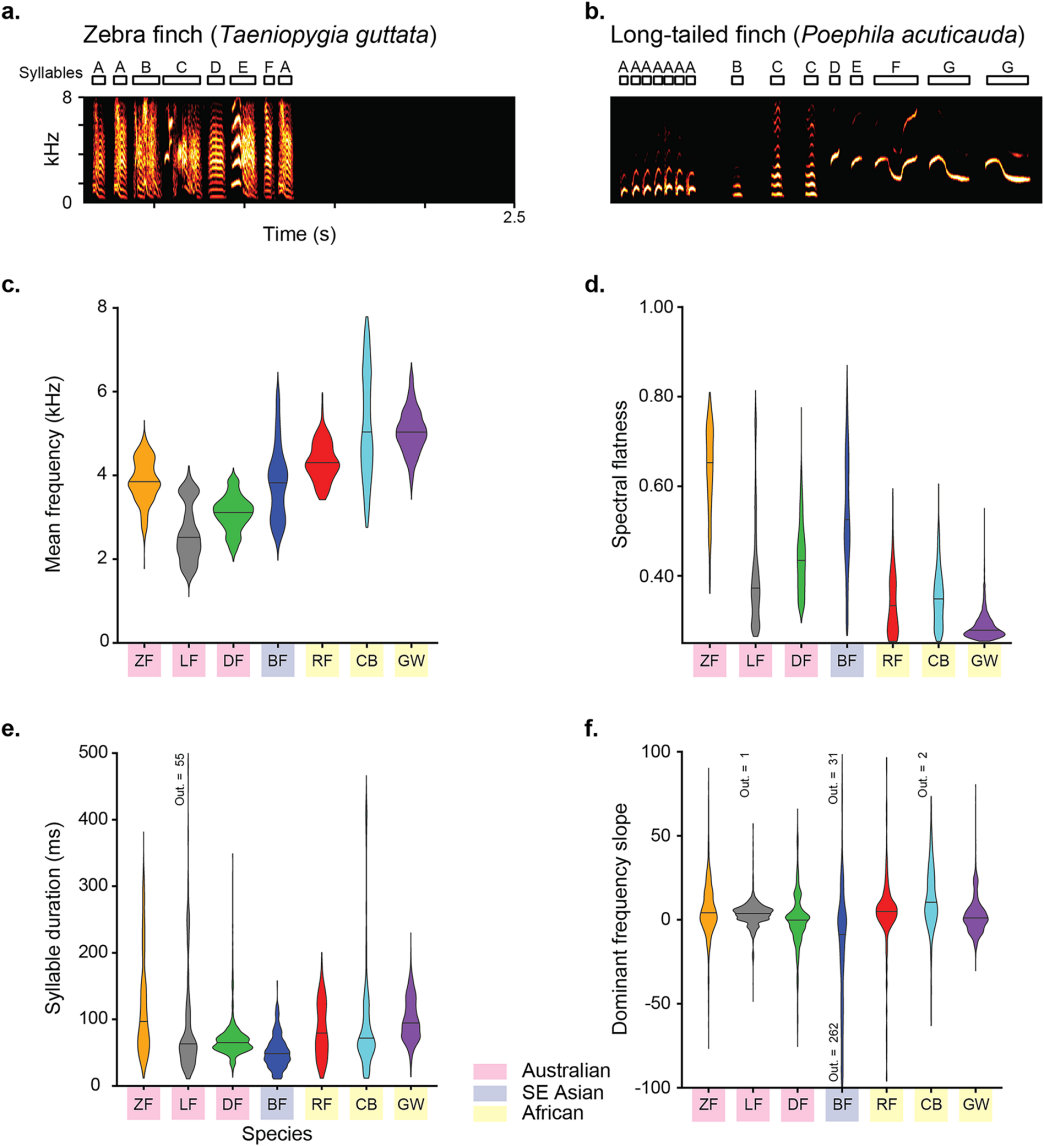
Sample spectrograms and acoustic measures for estrildid song syllables. (**a**) ZF song syllables are predominantly ‘broadband’ with multiple harmonic bands and “noisiness” between those, (**b**) while LF songs contain characteristically “clean” and tonal syllables. Violin plots show the distributions of 4 acoustic features that were measured from each species song for comparison. Features shown here were selected to provide contrasts for the degrees to which species may be similar or different based on the specific acoustic features considered. Horizontal line across each violin shows the distribution’s median value. (**c**) Mean frequency distinguished the songs of Australian (M ± SD = 3050 ± 661 Hz), Southeast Asian (M ± SD = 3847 ± 882 Hz), and African (M ± SD = 5031 ± 872 Hz) species. (**d**) Spectral flatness differed between African songs and those of other species except LFs. (**e**) Syllable duration measures overlapped among species, but the distributions of syllable duration for each species differed. (**f**) Dominant frequency slope, a spectrotemporal feature, differed little by species. The number of syllables outside of the plotted range is specified for each species.

The mating vocalizations (songs) of songbird species in the family *Estrildidae* (estrildid finches or waxbills/grassfinches) differ widely in spectral and temporal features, despite similarities in evolutionary origin, size, habitat, and diet^9,13,28–33^ (Figs. 1 and 2). In each species, song syllables are acoustically complex, species-specific, and learned, providing a rich diversity of signal features for species and individual recognition. Across species, songs are composed of hierarchically-organized acoustic units; notes are grouped to form “syllables,” and sequences of syllables form song^32–39^. Quantitative studies of estrildid song have largely focused on one or two species^13,30–33,36–38,40^, and either composite measures^41–44^ or individual features, such as fundamental frequency^45,46^.

Here, we measured 21 acoustic features of each song syllable, and used dimensionality reduction and machine learning classifiers, including multivariate, unsupervised and supervised models, to identify acoustic features that accurately assign song syllables to each of seven estrildid species. Results of these approaches were used to quantify and compare song acoustics across species and identify features that scale with phylogenetic distance to identify the acoustic features that predict species identity. Identifying and understanding these features will inform comparative behavioral and neural research and produce a song-evolutionary framework for the study and interpretation of processes and mechanisms underlying communication perception, selectivity, and preference^8,13,47^. We quantified song syllable acoustics in 3 Australian species, 3 African species, and 1 Southeast Asian species, all with known phylogenetic relationships^48–51^. We predicted that basic frequency (e.g., fundamental frequency) and power distribution features (e.g., measures describing the distribution of energy across frequencies such as kurtosis) would differ most across species and least within a species, consistent with previous spectral categorizations^13,20,29,33,52^. Further, we predicted that species would show less differentiation in spectrotemporal features due to the diversity in spectrotemporal modulation across syllables and species^53,54^. We also predicted that features known to be used by birds for sound discrimination in behavioral studies (e.g., fundamental frequency, Weiner entropy, time entropy, frequency modulation)^31,55–57^ would be the most important in syllable classification. Additionally, we predicted that song syllable classification accuracy would scale with acoustic similarity between species, such that greater overlap in acoustic feature space would predict greater misclassification errors in syllable labelling. Finally, we tested the hypothesis that evolution of frequency characteristics correlates with phylogeny, while power distribution and spectrotemporal features are labile and not constrained by phylogeny. To test this, we performed a phylogenetic signal analysis and quantified the effects of body size and phylogenetic inertia on song features.

## Results

### Frequency, power distribution, and spectrotemporal components explain 72% of variation in song

Our first set of analyses characterized the principal components (PCs) that allow us to represent song in reduced dimensional acoustic space, while explaining a majority of the variation between song acoustic features. Acoustic features were measured for the song syllables of 3 African species (red-cheeked cordon-bleu [*Uraeginthus bengalus*], CB; red-billed firefinch [*Lagonosticta senegala*], RF; gold-breasted waxbill [*Amandava subflava*], GW), 3 Australian species (double-barred finch [*Stizoptera bichenovii*], DF; long-tailed finch [*Poephila acuticauda*], LF; zebra finch [*Taeniopygia guttata castanotis*], ZF), and 1 Southeast Asian species (Bengalese finch [*Lonchura striata domestica*], BF). We conducted PCA using 21 feature values for each of the 15,081 syllables in our song dataset. The first 3 PCs explained 72.28% of the variation in syllable acoustics and represented the song frequency (PC1), power distribution (PC2), and spectrotemporal (PC3) features (Supplementary Table S1). Frequency features were singular frequency measures (e.g., fundamental frequency, mean frequency, dominant frequency). Power distribution features described the shape of the frequency power spectrum (e.g., entropy, spectral flatness, skew). Spectrotemporal features included syllable duration and changes in spectral features over time (e.g., modulation index, dominant frequency slope).

**Figure 3:**
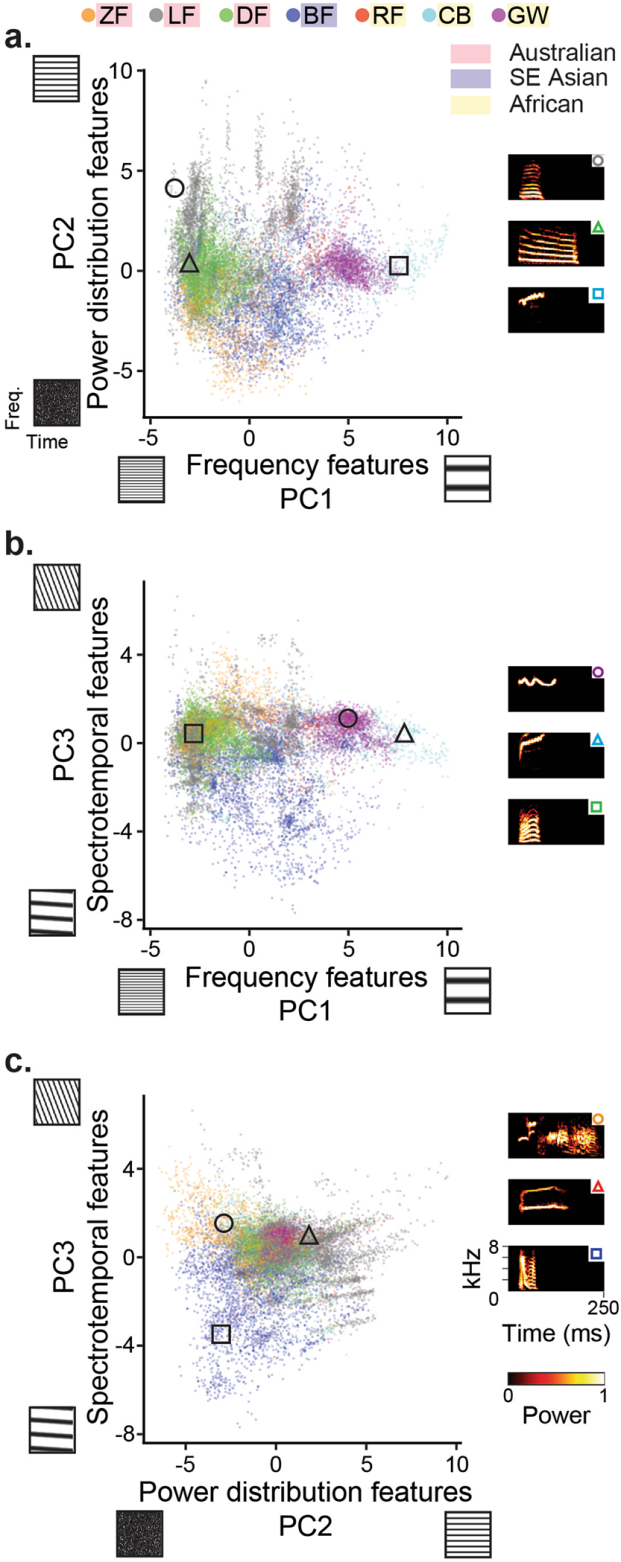
Principal component analysis (PCA) of all 21 acoustic features measured from each syllable. Syllables cluster within species (distinct clusters of the same color) and across species (muti-color clusters). (**a**,**b**) Syllables of African and Australian species clustered separately along PC1, with African species having higher values than Australian species, and the Bengalese distribution spanning the majority of the range between them. (**a**,**c**) The syllables of species with broadband species (ZF, DF, BF) and those with narrowband, tonal songs (LF, RF, CB, GW) clustered along PC2. (**b**,**c**) There are relatively uniform distributions along PC3, except for in the BF, whose distribution includes many low values in PC3. Spectrograms on the axis anchors are synthetic sounds that represent what each respective PC axis captures. These spectrograms show the distribution of energy across frequency (y-axis) and time (x-axis). Spectrograms to the right of each plot show real syllable samples from the respective plot. Symbols within the syllable spectrogram are color-coded according to species identity. Syllable locations within the plots are outlined in their respective shape (black outline used here for contrast). Overall, sample syllables confirm the distribution of energy expected for different positions on each graph (i.e., by combination of the axis coordinates).

The degree of shared and unique volume in PC space differed significantly across species comparisons (Fig. 3). Clusters in PC space represent single species (i.e., single color present; e.g., LF clusters around PC1 = − 3, PC2 > 2.5; Fig. 3a) or mixed species (i.e., multiple colors present; e.g., BF, DF, LF, ZF cluster around PC1 = − 2.5, PC2 = 0; Fig. 3a) syllables. Species differed in volume spread (e.g., GW syllables clustered in a small space and BF syllables were more variable as a species; Fig. 3b).

Species clustered into biogeographical phylogenetic groupings along the dimension of frequency components (Fig. 3a,b; Supplementary Fig. S1). Australian species (ZF, LF, DF) had low PC1 values, corresponding to low frequency vocalizations. African species (RF, CB, GW), on the other hand, had high PC1 values, corresponding to high frequency vocalizations. The BF frequency values ranged between the Australian and African species, with an intermediate median value and variance spanning the range of the other species. Syllables showed no clear phylogenetic clustering along power distribution (Fig. 3a,c; Supplementary Fig. S1). With respect to power distribution, ZF generally scored lowest, characteristic of vocalizations with a high spread in frequency and high entropy (e.g., noisy, broadband harmonic song). LFs, on the other hand, scored higher, characteristic of vocalizations with low harmonicity and low entropy (e.g., tonal song). All other species were intermediate between ZF and LF. Finally, species showed no clear phylogenetic clustering or differentiation in the spectrotemporal axis (Fig. 3b,c; Supplementary Fig. S1). BF syllables generally scored lowest in spectrotemporal component values, characteristic of vocalizations with short duration and down-sweeps. All other species scored similarly to each other.

### Species’ syllables differentially overlap in acoustic PC feature space along cladistic groupings

**Figure 4:**
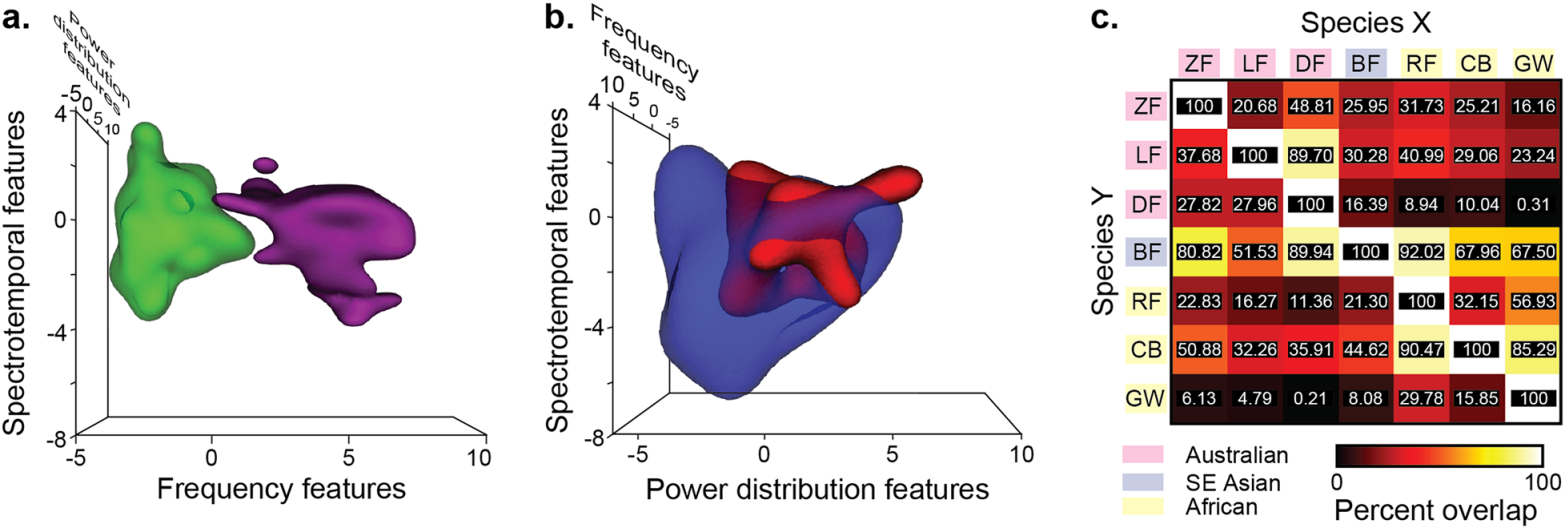
Overlap of volumes in PC space differed in pairwise species comparisons. (**a**) The feature volumes of DF (green) and GW (purple) syllables show minimal overlap (< 1%). DF songs are composed of harmonic syllables with low fundamental frequencies and slow spectrotemporal modulations, while GW songs have high-frequency, tonal syllables with fast temporal modulation. (**b**) BF (blue) and RF (red) songs have highly overlapping volumes. Specifically, 92% of RF acoustic feature space is shared with BFs. RFs share fast-to-moderate spectrotemporal modulations and variable entropy between harmonic banding with the BFs. RF syllables tend to be higher in frequencies, lower in harmonic band number, and have more upsweeps than BF syllables. (**c**) Hypervolume overlap indices quantifying the percent of Species X’s volume in Species Y’s volume. Note that values depend on which species in a pair is X and which is Y, and volume overlap can be asymmetric: a large proportion of Species X’s volume may overlap with Species Y, but a small proportion of Species Y’s volume may overlap with Species X’s volume.

Our next set of analyses quantified the amount of overlap between species in the reduced 3-dimensional acoustic feature space. Species boundaries were generated in the acoustic PC space using a support vector machine (SVM) algorithm (Fig. 4a,b). We obtained the Jaccard similarity index as a measure of the proportion of overlap between species in terms of the total space occupied by both (Supplementary Fig. S2). The greatest amount of shared volume (> 30%) occurred between CB and BF (37.02%) and between CB and RF (31.14%), while the smallest amount of overlap (< 5%) occurred between GW and DF (0.13%), GW and LF (4.13%), and GW and ZF (4.66%).

We also calculated both asymmetrical pairwise overlap indices for each species (i.e., what proportion of *species X* overlaps with *species Y*, and vice-versa; Fig. 4c). The largest overlaps were between RF in BF (92.02%) and RF in CB (90.47%), and the smallest overlaps were between GW in DF (0.31%) and DF in GW (0.21%). We plotted the volumes between RF and BF (Fig. 4a) and between GW and DF (Fig. 4b) as extreme examples of degree of overlap between species. Spearman correlation showed asymmetrical feature space overlap between species; large overlap of *species X* in *species Y* did not indicate large overlap of *species Y* in *species X* (asymmetrical), and these asymmetrical indices of overlap were not correlated (Spearman rank correlation: *r*_*s*_ = 0.055, *N* = 21, *P* = 0.81).

The overlap results highlight two key findings regarding phylogenetic relationships. First, species tend to overlap more with close relatives than distant relatives. This is shown by the higher overlap percentages (hotter colors in Fig. 4c) among Australian species (upper left corner) and African species (lower right corner). Second, a considerable amount of each species’ volume overlapped with that of BF song (hotter colors going across the BF row in Fig. 4c). This result reflects the broad range of acoustic features in BF syllables, which spanned the acoustic ranges of both the African and Australian species.

### Six acoustic features yield 93% classification accuracy of syllables to species

We next identified the acoustic features that were most informative for classifying syllables by species and tested the performance of classification models using those features. Results from the feature selection procedure showed that 6 features were most informative for explaining species’ differences in song: the minimum-, maximum-, and mean- fundamental frequency, spectral flatness, mean frequency, and duration of a syllable (see Fig. 2c–e and Supplementary Fig. S3).

Once we determined the 6 acoustic features that were most important for discrimination, we used those features to train and test a random forest model for syllable classification. The 6-feature model classified species syllables with 92.89% accuracy. Comparing the 6-feature model performance with the 21-feature model using their weighted F_1_ Scores showed no difference in performance (21-feature model: M ± SD = 0.97 ± 0.031; 6-feature model: M ± SD = 0.95 ± 0.050; two-tailed independent samples *t* test: *t*_12_ = 1.01, *P* = 0.33).

**Figure 5:**
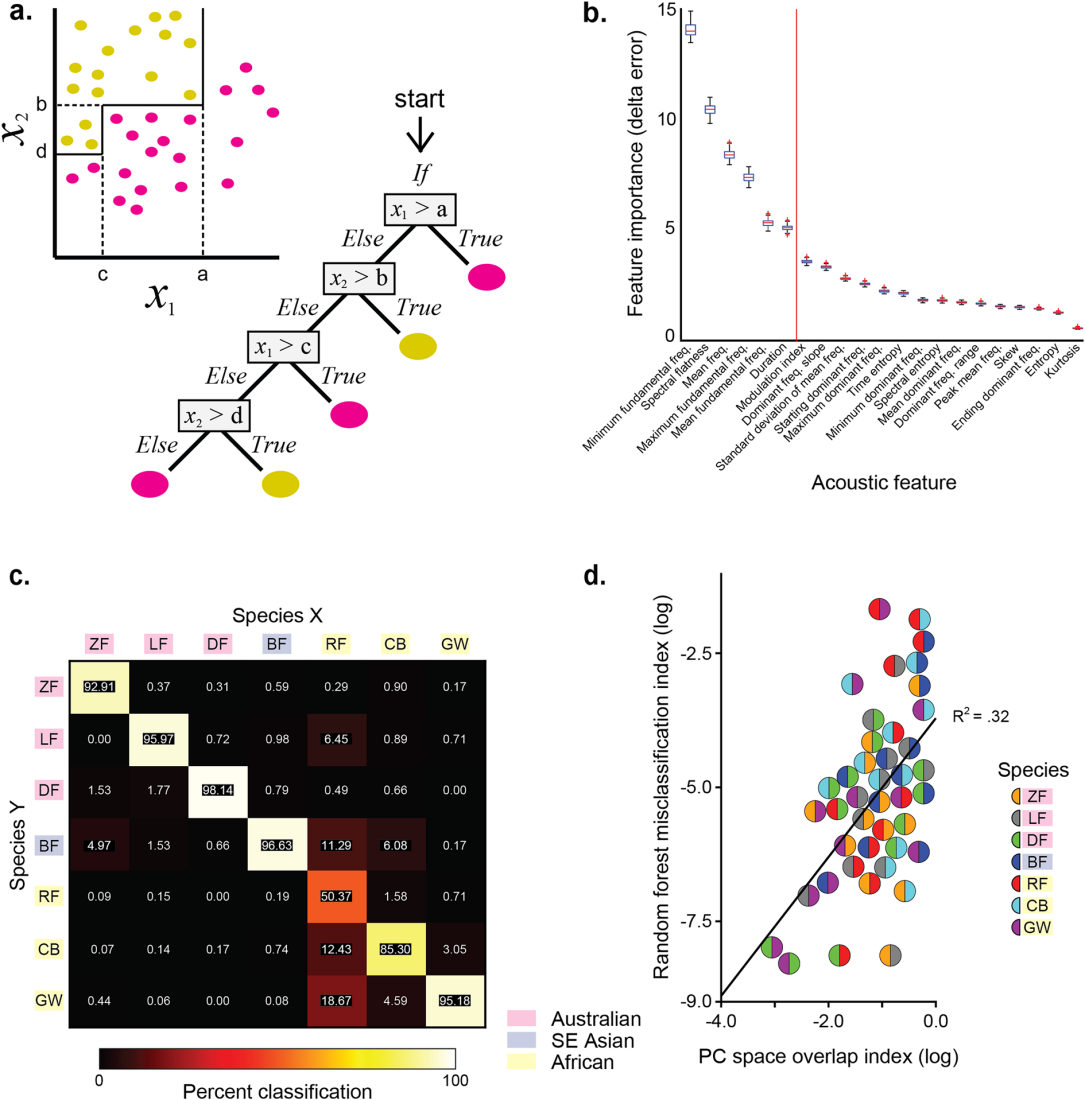
Classification of song syllables into species groups based on acoustic features (**a**) Sample flow chart of how decision trees (bottom right) create classification boundaries around data (top left). X1 and X2 represent two acoustic features along which the decision tree is learning the characteristics of each of the two hypothetical species (yellow and pink) in these dimensions. The classification is presented as a series of logical tests such that if *x*_*1*_ > *a* the syllable is labelled as belonging to species “pink”, otherwise the syllable advances to the next test, where if *x*_*2*_ > *b* the syllable is labeled as belonging to species ”yellow”, otherwise the syllable continues advancing through the tests until it meets a criterion for receiving a species label assignment. (**b**) Boxplots of delta error scores for each of the 21 features across 100 iterations of feature selection. Higher delta error scores correspond to higher feature importance in classification. We used the inflection point in delta error scores to identify the most informative features (those to the left of the red line at the inflection point) and used these in the random forest classification model. (**c**) Confusion matrix showing the percent of Species X syllables classified as Species Y by the 6-feature random forest model. Classification accuracy was > 90% for most species pairs. (**d**) Scatter plot showing that PC volume overlap between two species positively correlated with misclassification.

Similar to the hypervolume overlap analysis, the random forest analysis showed asymmetrical patterns of misclassification between species (Fig. 5c); misclassification of *species X* syllables as *species Y* was not indicative of misclassification of *species Y* syllables as *species X* (asymmetrical). Asymmetrical misclassifications were not significantly correlated (Spearman rank correlation: *r*_*s*_ = 0.38, *N* = 21, *P* = 0.091). Misclassification was highest for ZF (7.09%), CB (14.70%), and RF (49.63%) syllables, while misclassification was lower than 5% for all other species.

Because the total number of syllables differed across species, we next tested the effects of sample size on classification accuracy. Specifically, we tested classification accuracy with reduced sample sizes and with balanced sample sizes in which each species contributed the same number of syllables to the dataset. We conducted under-sampling and resampling procedures and ran the random forest classification on the under- and re-sampled datasets to compare classification performance with these datasets to that of our original dataset (Supplementary Fig. S4). Results from the under-sampled data showed that model performance significantly decreased with reduced sample sizes (F_1UndSamp_ (M ± SD) = 0.84 ± 0.088; two-tailed independent samples *t* test: *t*_12_ = 3.02, *P* = 0.011). For the 6 species that were under-sampled (i.e., all except RF), classification accuracy was lower with the under-sampled dataset than with the full dataset (Supplementary Fig. S4). For RF song, syllable classification was slightly (5%) better with the under-sampled dataset. Results from our resampled data suggest no change in model performance using the balanced sample sets (F_1SCUT_ (M ± SD) = 0.93 ± 0.032; two-tailed independent samples *t* test: *t*_12_ = 0.84, *P* = 0.42). In most species, classification accuracy changed only slightly (< 3%) with under- and over-sampling consistent with their SCUT resampling (e.g., over-sampled species decreased in misclassifications, under-sampled species increased in misclassification). We found inconsistent change with the resampling of GW and LF songs following resampling, but these were minor (1.3% increase and 0.7% decrease in misclassification, respectively). In contrast, results showed a modest decrease in misclassification of CB syllables (from 15 to 9%) and a large decrease in misclassification of RF syllables (from 50 to 17%). The large improvement in RF syllable classification suggests that sample size affects model predictions more for this species than for others. Overall, the results of sample size manipulations showed that the accuracy of random forest classification was robust to differences in sample sizes, with the exception of one species.

In order to test the relationship between acoustic PC space overlap and syllable misclassification, we measured the Pearson correlation on the log-transformed indices for each pairwise species comparison. We found a moderate correlation between acoustic space overlap and syllable misclassification (Pearson correlation: *r* = 0.57, *N* = 42, *P* < 0.001). The results show a relationship between the pair-wise quantification of similarity (i.e., PC volume overlap) and the multi-species quantification of similarity (i.e., random forest misclassification, wherein syllables may be classified as any of the seven species), such that greater overlap in acoustic component space is indicative of greater syllable misclassifications between species (Fig. 5d). For example, the highest misclassification of RF syllables occurs with the other African species (i.e., CB and GW) and with the BF (Fig. 5c), consistent with high overlap in PC space between RF and these species (Fig. 4c).

### Frequency features correlate with phylogeny

**Figure 6:**
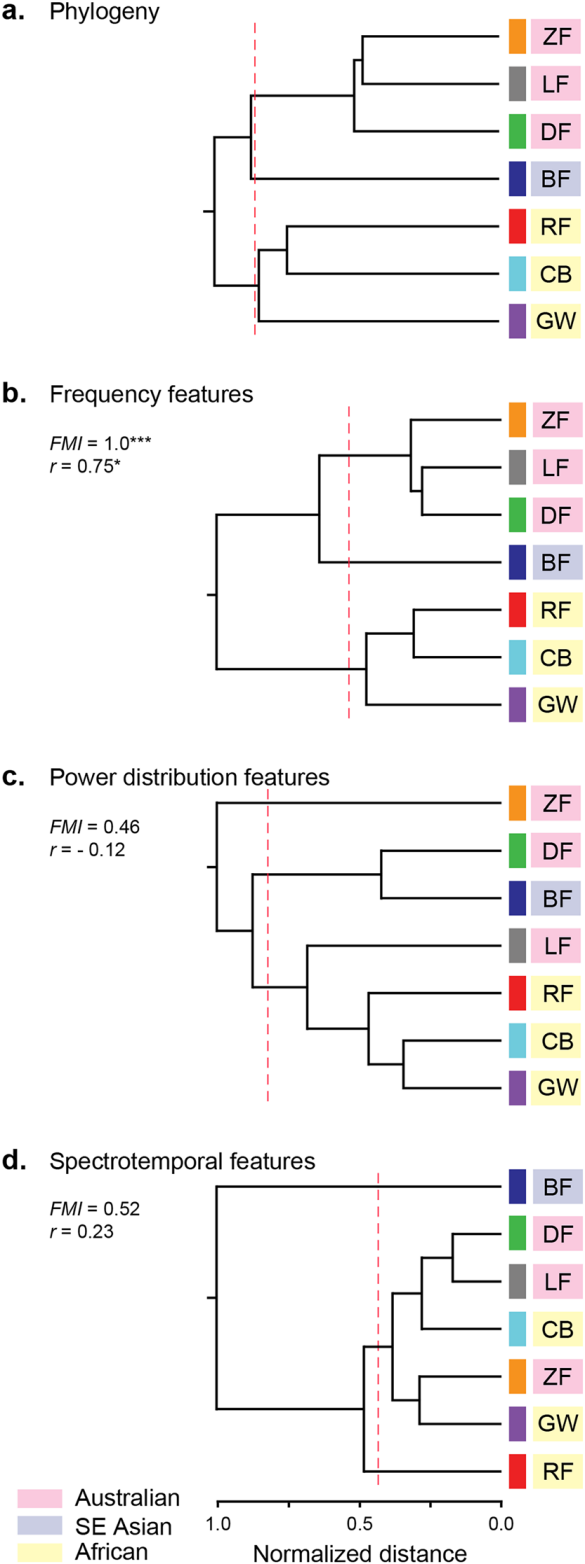
Dendrograms comparing phylogeny and acoustic similarity. Dashed red lines indicate where along each tree k = 3 clusters for FMI calculation. All distances were normalized to range between 0.0 (no distance) and 1.0 (most distant) for distance comparisons using acoustic features and phylogenetic relatedness. (**a**) Phylogenetic tree for the 7 species. (**b**) Frequency features (PC1) clustered species according to their biogeographical clades (FMI = 1.0, P < 0.001): Australian, Southeast Asian, and African. EM-Mantel procedure found significant phylogenetic signal in PC1 (r = 0.75, P = 0.015). (**c**) Power distribution features (PC2) did not cluster species according to biogeographical clades (FMI = 0.46, P > 0.05) nor express phylogenetic signal (r = − 0.12, P > 0.05). (**d**) Spectrotemporal features (PC3) did not cluster species according to their biogeographical clades (FMI = 0.52, P > 0.05) nor express phylogenetic signal (r = 0.23, P > 0.05). Significant results denoted by asterisks at their corresponding alpha levels (* < 0.05, ** < 0.01, *** < 0.001).

Finally, we identified the acoustic features that correlated with species relatedness. Comparing the topologies of the dendrograms for acoustic and phylogenetic distance revealed that species clustered along frequency features consistent with their geographical distributions (i.e., Australian, African, and Southeast Asian; *FMI* = 1.00, *P* < 0.001; Fig. 6b). Post-hoc FMI tests on the acoustic features that load significantly onto frequency component (PC1) found significant similarity between biogeographical groupings and syllable starting dominant frequency (*FMI* = 0.73, *P* = 0.042). No other acoustic feature showed significant shared topology with the phylogeny (all *P* > 0.05; see Supplementary Table S1).

Clusters along power distribution (*FMI* = 0.46, *P* = 0.15; Fig. 6c) and spectrotemporal (*FMI* = 0.52, *P* = 0.13; Fig. 6d) components did not match the species phylogeny. Within power distribution features (PC2), tonal species (LF, RF, CB, GW) formed one cluster, while DF and BF formed a second cluster, and ZF formed its own cluster. The distinction between these clusters may involve harmonic banding (tonal vs. BF-DF clusters) and amount of noisiness (e.g., entropy, flatness) between harmonics (highest in ZF vs lower in all other species). Within spectrotemporal features (PC3), the BF cluster likely resulted from the large proportion of short duration syllables in the song repertoire of this species, while the RF cluster is due to the large proportion of longer-duration syllables in their song repertoire (as opposed to smaller proportions of song repertoire represented by very long syllables in LFs and CBs). All other species (intermediate between these two extremes) clustered together.

Results from the standard Mantel test demonstrated a significant correlation between phylogenetic distance and frequency (*P* = 0.006), but not power distribution (*P* = 0.645) or spectrotemporal (*P* = 0.310), components. The Brownian motion EM-Mantel (one-tailed) test detected a significant phylogenetic signal in frequency components (*r* = 0.75, *P* = 0.015). The result suggests acoustic frequency relationships have evolved as predicted by the evolutionary time between species. Despite significant and robust omnibus results, post-hoc Mantel tests on the acoustic features that load significantly onto the frequency component (PC1) did not detect significant correlations between phylogenetic distance and any of the individual features (all *P* ≥ 0.058; see Supplementary Table S1). To measure the relationship between frequency features and body size while controlling for the effect of phylogeny, we performed Mantel tests with phylogenetic permutations (ppMantel) on the distance matrices for PC1 (frequency component) and body size morphometrics (body mass and tarsus length). Results from the ppMantel tests found significantly positive correlations between the song frequency component and both tarsus length (*r* = 0.21, *P* < 0.001) and body mass (*r* = 0.25, *P* < 0.001) after controlling for phylogenetic relatedness. We quantified the phylogenetic inertia in each acoustic feature and component to determine the degree to which acoustic feature evolution is constrained by phylogeny (Supplementary Table S1). Results showed that frequency features were moderately to highly conserved according to phylogeny (e.g., phylogenetic inertia for PC1 = 57%), while power distribution and spectrotemporal features showed low phylogenetic constraint (i.e., phylogenetic inertia for PC2 = 2%, PC3 = 5%; Supplementary Fig. S5). Comparing the effect sizes for the EM-Mantel test to the ppMantel tests revealed that the differences in frequency features are better explained by phylogenetic inertia (i.e., larger EM-Mantel correlation coefficient) than by body size.

## Discussion

We used computational models and direct measures of song acoustics to determine the features that accurately classify vocal communication signals by species, and to identify features that correlate with phylogenetic relatedness. By comparing the results of multiple approaches, we developed a framework for evaluating how species differ in vocal acoustic features and how these acoustic differences may have evolved. Results expand previous research and add comparative data from estrildids, supporting the conclusions that: (a1) song syllables can be described and compared by their frequency, power distribution, and spectrotemporal features; and (a2) these complex acoustic features vary within species and overlap in varying degrees between species. Novel findings are that: (b1) syllables can be accurately classified by species using six acoustic features; and (b2) simple frequency features map onto phylogeny and have phylogenetic inertia, suggesting that changes in these features are constrained by phylogeny. Previous neurophysiological and behavioral studies suggest that songbirds are sensitive to complex acoustics and manipulations in the relationship between acoustic features^31,55,58^. Generalization of findings to different species, however, has been criticized given the limited number of species and acoustic features typically studied, and the limited consideration of interactions between acoustic features in previous research^59^. Our paper aims to provide a standardized approach for future researchers to quantify the acoustic similarities between species, identify the acoustic features that optimize species-level classification, and determine which acoustic relationships may be associated with phylogeny. This song-evolutionary framework may be used to test the role of acoustic and phylogenetic relationships on species recognition in comparative studies.

Overall, our results show that estrildid songs can be meaningfully characterized in reduced acoustic dimensions from their frequency, power distribution, and spectrotemporal components (Fig. 3). Hypervolume analyses showed that species occupy specific regions of acoustic feature space and exhibit variable amounts of overlap with each other, representative of the acoustic similarity in their song syllable PC features (Fig. 4)^3,60^. In comparing species relationships along these components, we found that lower overlap in the three dimensions correlates with greater differentiability of syllables by their raw acoustic features (Fig. 5d). Phylogenetic analysis showed that clustering in frequency features follows cladistic groupings: Australian species cluster in the lower range of PC1 (i.e., these species generally produce lower pitch songs), African species in the higher range, and the Bengalese finch spans between these (Fig. 3a; Supplementary Fig. S1). Clustering in PCs 2 and 3, however, does not follow cladistic grouping.

Ensemble tree procedure for feature selection and model optimization showed that fundamental frequency (minimum, maximum, and mean), mean frequency, spectral flatness, and syllable duration are the features most important for classifying song by species. Most of these features (i.e., fundamental frequency; “entropy,” but see note on spectral flatness below) have been previously established for use in calculating similarity between songs for measurements of song imitation, variability, and change over time and experimental manipulations^41^. Spectral flatness is a measure of noisiness in the frequency domain of a signal, similar to spectral entropy^61^ and Wiener entropy^41^. Mean frequency has previously been found to vary along genetic lines (i.e., across species^31^ and strains^52^), but not rearing conditions when cross-fostered. These findings suggest that mean frequency could be used in classification by allowing variability in song within species ranges while still preserving species identities^62^. Previous studies with estrildids have not used duration as a diagnostic feature for species identity, despite its usefulness in categorizing vocalizations in song-^63^ and non-song-learning^64^ birds. Estrildid studies, however, have found that duration could be used in conjunction with other features to differentiate between syllables that otherwise overlapped in measures of entropy and fundamental frequency^65^. Fundamental frequency has also been shown to be used in categorical discrimination of vocalizations in estrildids^63^. Previous studies have also attributed variations in neural^45^ and behavioral^63^ response to these acoustic features in songbirds. Overall, the features identified as most important in song classification in our study parallel those previously identified in acoustic discrimination and preference research. Machine learning methods may be informative in further identifying the acoustic features that elicit species-recognition and neural tuning^66^.

Phylogenetic signal analysis provides a measure of the amount of trait variation that can be explained by evolutionary relatedness^30^ and has been used in species-level analyses of vocalizations across taxa, including birds^25,67–70^, anurans^71^, and mammals^72^. Acoustic features that identify species from field recordings allow measurement of biodiversity, migration, and habitat use^73–76^. Our analyses detected phylogenetic signal in frequency, but not power distribution or spectrotemporal, components. Despite song being a learned individual behavior, previous studies suggest that frequency features of song are strongly heritable and evolve in populations via natural selection^54,77^. Spectrotemporal characteristics are proposed to be less constrained due to the vocal production organ (i.e., the syrinx), which may provide a ubiquitous and flexible mechanism on which processes of cultural transmission (e.g., song learning) and sexual selection impose demands for faster rates of change^3,77,78^. Our results match previous studies in *Regulus* songbirds that suggest that phylogenetic signal in frequency features (PC1 in our study) may indicate constraint on these features by morphological structures involved in (or associated with) vocal production, while lack of phylogenetic signal in frequency bandwidth and modulation (related to PCs 2 and 3 in our study, respectively) suggest that these features are learned acoustic parameters^24^. Although significant differences exist in syringeal morphology across birds^25^, song learning in oscine birds—including estrildids—has been facilitated by an adaptable capacity for vocal production by conserving a uniform syringeal morphology that can meet the demands of culturally transmitted song^78^ (but see^25,54^ for discussions on constraints).

Previous electrophysiological studies have found neural tuning to power distribution (PC2) and spectrotemporal (PC3) modulations—but not to basic frequencies (PC1)—in regions critical for song learning (e.g., caudomedial nidopallium^31^, and L3^55^. Neurophysiological studies have characterized spectrotemporal tuning in auditory neurons and its role in song learning and perception^31,58,79,80^. Moore and Woolley^31^ found that neural selectivity for the spectrotemporal modulations is shaped by vocal learning during development, while basic frequency tuning measured from tone receptive fields is not. This study supports the hypothesis that basic frequency features are more conserved across individuals of the same species than are complex features^3,54^. The vocal acoustics captured by PC1 were more similar between closely related species than between distantly related species (Fig. 3a; Supplementary Fig. S1), suggesting that frequency features can be used to identify species and predict relatedness. This relationship between frequency features and phylogeny may function in species recognition, without visual or direct contact between signalers and receivers^81^.

Significant phylogenetic signal using the EM-Mantel procedure suggests that divergence in those features (i.e., PC1) may be accounted for by the simple “random walk” of traits as a function of the evolutionary time between species (i.e., Brownian motion). The absence of phylogenetic signal, however, does not necessarily mean that traits evolve independently of evolutionary constraints. Power distribution (PC2) and spectrotemporal (PC3) features, which did not express significant phylogenetic signal under Brownian motion, may instead be evolutionarily labile and change under alternative selection pressures and processes within species^3,54,77,78^, specifically those important for vocal communication (e.g., individual recognition, mate attraction, intrasexual competition, and cultural evolution)^13,25,62,82^. In this case, we would expect species to cluster along PCs 2 and 3 independent of their phylogenetic relatedness, and this result can be seen in the polyphyletic clustering of the species as well as in the divergence within the Australian (and to a lesser extent the African) species along PCs 2 and 3 (Fig. 3c; Supplementary Fig. S1). In the absence of significant phylogenetic signal, traits may be associated with alternative models of evolution that are not constrained to Brownian motion (e.g., adaptation)^83^. Our estimates of phylogenetic inertia provide further evidence that power distribution and spectrotemporal features are minimally constrained in their evolution (i.e., low phylogenetic inertia), suggesting that these features may reflect the plasticity needed to alter song features for inter- and intra-specific competition. Analyses using ppMantel tests showed that body size is correlated with frequency features in our species, a common finding in animals including birds^53,84,85^. Previous studies suggest that changes in frequency features may be constrained to changes in morphological features associated with body size such as size of the organs that produce (e.g., syrinx) or modulate (e.g., beaks) vocalizations^84^. Relatively small effect sizes for the frequency-to-morphology correlations, however, may suggest that differences in frequency characteristics may be further explained by factors other than the evolutionary changes in body or organ size. Previous studies in songbirds have suggested that learning may weaken (or even eliminate) the association between song frequencies and body or syrinx size^85,86^. We adopted a procedure for comparing effect sizes for the Mantel tests between frequency features, body size, and phylogeny. Comparing effect sizes from the EM-Mantel correlation to the ppMantel correlations for PC1 gives us an understanding of the relative importance of phylogenetic inertia in the evolution of frequency features^83^. Larger effect sizes for the ppMantel tests over the EM-Mantel would suggest that the correlations between frequency features and body size are not only due to phylogenetic inertia and there may be a direct link between the two traits. Overall, we found evidence of phylogenetic inertia (57%) in frequency features. Our results suggest that species’ relationships in frequencies features are predicted by phylogeny as opposed to adaptive processes such as convergent evolution. The relatively larger effect size for the EM-Mantel test over the ppMantel tests further implies that there is no direct causal link between body size and frequency features in these species^83^. Future studies may explore the differences in syringeal morphology and physiology as well as in song learning and copying-fidelity between species in order to further disentangle the role of genetic and cultural mechanisms on vocal feature evolution in Estrildids^87^.

Songbird vocalizations offer a diverse system for studying the effects of learning and evolutionary processes on behavior. Communication signals allow for coding, transmittance, and interpretation of social information between senders and receivers, and behavioral and neural studies in various animal taxa support this theory^1,13,20,58,88–90^. We therefore expect acoustic differences between species’ songs to scale with differences in perceptual mechanisms and to—in turn—affect processes of behavioral response, reproductive success, and ultimately speciation in estrildid finches^3,6,9,13,19,20,54,91,92^. The machine learning and statistical models used here identified the acoustic dimensions that best characterize and distinguish species’ songs. We also found that frequency features such as fundamental frequency correlated with phylogenetic distance, while power distribution and spectrotemporal features did not. These findings suggest that basic frequency characteristics and more complex characteristics are generated by different mechanisms. Selection may conserve frequency features that identify the species of a signaler^26^, while features such as power distribution and spectrotemporal modulations may vary across conspecific individuals and overlap between species^3^. These more complex and variable features may function to aid in individual recognition and competition for mates within species^93–95^. Diversity in syllable power distributions and spectrotemporal features may also be driven by interspecific acoustic competition and provide a mechanism by which sympatric species—whose acoustic frequency features have otherwise been conserved for interspecific communication—may vary and compete within their niche^96^. Further studies on the vocal acoustics in related evolutionary lineages will further disentangle the effects of genetics and learning on vocal communication and guide our understanding of the mechanisms driving communication production and perception. Future studies should incorporate measures of syntax (the temporal sequencing of syllables) into quantitative analyses of species difference in song acoustics.

## Methods

### Ethics statement

All animal handling and use procedures were conducted under the guidelines and approval of the Institutional Animal Care and Use Committee (IACUC) of Columbia University. The study reported here was conducted in compliance with the recommendations in the ARRIVE guidelines.

### Experimental model and subjects

We analyzed the acoustics of songs recorded from males in each of 7 estrildid species. Species were selected based on differences in song acoustics and phylogenetic relatedness^25,48,97^ (Fig. 1). The songs of 3 African species (red-cheeked cordon-bleu [*Uraeginthus bengalus*], CB; red-billed firefinch [*Lagonosticta senegala*], RF; gold-breasted waxbill [*Amandava subflava*], GW), 3 Australian species (double-barred finch [*Stizoptera bichenovii*], DF; long-tailed finch [*Poephila acuticauda*], LF; zebra finch [*Taeniopygia guttata castanotis*], ZF), and 1 Southeast Asian species (Bengalese finch [*Lonchura striata domestica*], BF) were studied. Previous studies have reported differences between the Bengalese finch and its wild type, the white-rumped munia (*Lonchura striata*), in body size and song syntax (temporal sequencing of syllables)^36,98^. Studies comparing the syllable acoustic features in the two strains have reported differences only in frequency modulation^36^. We note this and that the methods for calculating frequency modulation in those studies is operationally different from the calculation of modulation index measure in this study. All birds were housed in our lab in mixed-species colonies maintained at 27 °C, 30% humidity, and a 14–10 light/dark cycle with ad libitum access to food and water. Sound recording chambers were maintained at the same conditions.

### Song recording and syllable extraction

Adult (> 120 days post-hatch) male finches were individually placed in sound-attenuating chambers (MAC-1, Industrial Acoustics Company) equipped with microphones (MKE 2-60, Sennheiser) for song recording using Sound Analysis Pro Recorder (v2011; 44,100 Hz sampling rate)^41^. Individuals were continuously recorded for 1–3 days. All files were filtered for instances of singing and verified song files were stored for analysis. Files were pseudo-randomly selected in order to ensure proportionally equivalent sampling between and within recording days, and the representation of the repertoire diversity within individuals. For each species, 20 song motifs recorded from each of 10 birds were analyzed (i.e., 200 motifs per species), except when limited by availability. Songs of 4 CBs (80 total motifs for CB) and 3 RFs (for which one male only produced 8 motifs; 48 total motifs for RF) were analyzed. For GWs, in which motif structure is not clearly defined, we used uninterrupted bouts of song syllables (as described in^33^) with inter-syllable intervals (ISI) < 2500 ms (ISI, Median ± IQR = 842.38 ± 579.37 ms; Number of syllables per bout, Median ± IQR = 8 ± 5 syllables). Hereafter, procedures described for “motifs” apply to these bouts.

Song motifs were bandpass filtered (250–8000 Hz) and root-mean-square power matched (65 dB) prior to analysis. Motifs were segmented into syllables using the warbleR library^61^ for R (v. 4.0.3)^99^. Syllable boundaries were visually inspected and adjusted as needed to ensure accurate segmentation. The complete dataset consisted of 15,081 song syllables with the following number of syllables for each species: BF: 3993; CB: 883; DF: 4322; GW: 1765; LF: 2821; RF: 207; ZF: 1090.

### Data preparation

Acoustic measurements were made from each syllable using the *specan* function in warbleR, which relies on the seewave package^100^. We measured 21 features to quantify spectral, temporal, and spectrotemporal acoustics of each syllable (Supplementary Table S1). Due to the diversity of syllable types, including syllables with minimal acoustic structure (i.e., resemble white noise), 3 of the 21 features could not be measured from some syllables (6.22% of all syllables per feature); warbleR did not detect a fundamental frequency for these syllables. In order to include these syllables in our analyses, estimates of these missing data (i.e., minimum-, maximum-, and mean- fundamental frequency) were imputed through regularized iterative principal component analyses (riPCA) for each species using the missMDA package^101^ for R. riPCA provides estimates of the missing values by taking into account the similarities between syllables (within species) and the relationships between acoustic features^102^. Projection of the imputed data dimensions onto the incomplete data’s PCA dimensions suggested no significant deviation from the reference dimensions, and therefore no undue influence of the imputed data on the completed dataset. We therefore use the imputed data set in all subsequent analyses.

### Principal component and volume analysis

Given the high number of measured features and variation in multiple features^103^, we performed principal component analysis (PCA) on the 21 acoustic features for the 15,081 syllables in our song dataset using the *prcomp* function in R^99^. PCA projects the distribution of syllable features into low-dimensional space and reveals the major underlying axes of variation in syllable acoustic structure. We used a feature loading criterion^104^ [*L*_*Crit*_, Eq. (1)] to identify the raw acoustic features that significantly loaded to each principal component (PC; Supplementary Table S1) as follows:

1 $${L}_{Crit}= \sqrt{\frac{1}{f}} ,$$

where *f* is the number of raw acoustic features used in PCA. We found that the first 3 PCs met Kaiser-Guttman criterion^105^ (i.e., Eigenvalues > 1), included significant loadings by all 21 of the raw features, and explained 72.28% of the variance in the data. We used these first 3 PCs in subsequent analyses. Using *L*_*Crit*_, we explored the qualitative relationship between acoustic features that significantly loaded to each PC.

We next quantified and compared the acoustic space occupied by each species’ song syllables. We used the support vector machine (SVM) method in the hypervolume package^106^ for R to determine the boundaries of each species’ song in 3-dimensional PC space. SVM is a nonparametric classification algorithm that estimates the nonlinear one-class boundaries (e.g., *species X* vs. not *species X*) in the space of the original data^107^. Importantly, SVM generates the boundaries of acoustic space for each species that includes all data points, regardless of distribution of points within the space (Fig. 4a,b and Supplementary Fig. S2). This is in contrast to volumes estimated based on central tendencies or centers of mass^42,107,108^.

Using these species boundaries, we quantified species volume and pairwise overlap between volumes using the Jaccard similarity index calculated by the *hypervolume_overlap_statistics* function^106^. The Jaccard similarity index is a measure of the proportion of shared space (i.e., intersection volume) to total space (i.e., union volume) occupied between two species. In order to quantify the proportion of shared-to-unique volume in acoustic feature space between species (e.g., what proportion of *species X*’s distribution is within *species Y*, and vice-versa) we calculated directional overlap indices between each species pair. For these indices, we divided the shared volume by the focal species’ volume. We used Spearman correlation to test whether the overlap between species was symmetrical (i.e., if large overlap of *species X* in *species Y* was indicative of large overlap of *species Y* in *species X*). Because of the random initialization of parameters during the SVM algorithm^107^, within-species volume boundaries varied slightly across computations. Therefore, SVM volume boundaries and overlap indices were computed as above, stored, and averaged across 100 iterations.

### Feature selection and random forest classification

After quantifying acoustic similarity between species using volume overlap in low-dimensional space, we tested which acoustic features were most relevant for discriminating one species’ syllables from others. We used a random forest algorithm in order to test the accuracy of syllable classification to a species using the 21 acoustic features^62,109^. Random forest classification was conducted using the *TreeBagger* function in MATLAB^110^, which implements Breiman’s algorithm^111^, to create 1000 decision trees. Each decision tree in the random forest receives a subsample of syllables and acoustic features in the dataset, and learns a flowchart-like series of binary decisions to assign a species label to each syllable (see Fig. 5a). Importantly, species’ syllables were sampled for training with probabilities calculated by taking the average number of syllables per motif, per species. This standardization controls for the effects of unbalanced sample size on model performance by allowing the random forest to train and test itself using syllable proportions that are reflective of the natural occurrence in species song and not of the sampling effort in our study. Each decision tree in the random forest then casts a “vote” on the species label for each syllable, and the syllable is labeled according to the majority vote. *TreeBagger* uses two-thirds of the input data in order to build decision trees and train the classification model, and then tests model performance using the remaining one-third of the data.

We next tested the importance of each acoustic feature for accurately classifying syllables to species (Fig. 5) using *TreeBagger* with specified parameters to optimize feature selection. In this process, each tree was given the complete set of acoustic features to use in its decisions and sampled species’ syllables with equal probabilities. Splitting decisions at each node of the tree were made after accounting for predictive association between features. Specifically, the algorithm took into account the interaction between features as well as all other alternative feature splits possible at each node. Feature importance was computed by measuring the change in prediction error (“delta error”) that results if values of a given predictor are randomized (Fig. 5b and Supplementary Table S1). As such, predictors that are more important to maintaining classification accuracy would yield higher delta error values. *TreeBagger* calculates a single importance (i.e., delta error) value for each predictor across all decision trees. Like SVM, *TreeBagger* relies on the subset of data used to train the classification model. We therefore employed a custom script (SONg-TUUL) to compute, store, average, and plot the bootstrapped model metrics for 100 iterations of feature selection. We rank-ordered the importance scores for features and identified the inflection point^105^ to find the features that were most important for distinguishing the syllables of species’ song. Our analysis showed that the first 6 rank-ordered features scored significantly higher than the rest of the acoustic features.

In order to test the efficacy of the classification model using only the 6 most important features discovered (“6-feature model”), we conducted 100 iterations of random forest training and testing using just these features as first described for the 21 features (“21-feature model”). We compared performance of the 6-feature model to the 21-feature model using the weighted F_1_ Scores for each^112^. The F_1_ Score takes into account model sensitivity (the proportion of all syllables produced by birds of *species X* that were correctly classified as belonging to *species X*) and precision (the proportion of all syllables classified as belonging to *species X* that were actually produced by birds of *species X*) and yields an index value between 0 and 1 for each species. We calculated the weighted average of this score across species (using number of syllables per species in our dataset as the weight) to obtain the model-wise F_1_ Score for each of the random forest classification models. We tested the difference between the 6-feature and 21-feature models’ F_1_ Scores across iterations using a Student’s independent samples t-test.

Because the number of syllables in the datasets for each species differed, we tested the effect of sample size on model performance. For this, we generated syllable datasets in which sample sizes were matched across species by under-sampling and resampling, and then compared their model-wise F1 scores from the random forest classification to that of our originally-sampled data, using the 6-feature model for all three datasets. First, we generated an under-sampled dataset by randomly selecting 207 syllables without replacement from the full dataset for each species other than RFs to match the RF sample size. Second, to test whether the model—and specifically the low-sample species—would improve classification with increased sample size, we generated a resampled dataset by applying a multi-class synthetic minority oversampling technique (SMOTE) with cluster-based under-sampling using the scutr package^113^ in R; the complete procedure is referred to as SCUT^114^. The SCUT procedure balances multi-class data by first dividing the total sample size (15,081 syllables) by the number of classes (7 species) to calculate a balanced target sample size of 2154 syllables for each species. Species that have sample sets larger than the target sample size are under-sampled by iteratively removing same-species syllables with the smallest distance to its neighbors. Species that have sample sets smaller than the target sample size are over-sampled through SMOTE procedure^115^. Briefly, SMOTE artificially grows sample sets by creating simulated values along the feature distance vector between nearest neighbors in the original dataset. As such, simulated values are confined to the original data’s region in multidimensional feature space, and these “synthetic” values prevent classification models from over-fitting on the (repeated) minority class samples as is done following traditional over-sampling techniques (e.g., resampling with replacement)^115^.

### Transformation and association between random forest misclassification and PC volume overlap

Because accurate syllable classification depends on the discriminability of each species’ syllable acoustics, we tested whether random forest performance using the discovered features correlated with the amount of overlap between species in acoustic PC space. Similar to the PC volume overlap indices, we calculated the asymmetrical pairwise misclassification indices from random forest as “the number of syllables belonging to *species X* that were incorrectly classified as *species Y*.” In order to test the association between the acoustic similarity of species’ song syllables in PC space and the discriminability of those same syllables based on the most important acoustic features discovered, we plotted and conducted a Spearman test on the log-transformed values of these indices. To account for indices equal to 0 (i.e., random forest misclassifications between GWs and DFs), we added a constant following Stahel’s quantile recommendation^116,117^ before log-transforming. Stahel’s recommended adjustment calculates the constant by dividing the squared first quantile by the third quantile of the data (excluding 0 s). The transformed hypervolume overlap (*T*_*HY*_) and random forest misclassification *(T*_*RM*_) indices were calculated as follows:

2 $${T}_{HY}= \mathrm{log}(H+ 0.05389)$$

3 $${T}_{RM}= \mathrm{log}(R+ 0.0004216)$$

where *H* is the original hypervolume overlap index and *R* is the original random forest misclassification index between species. Finally, we plotted and used the transformed pairwise PC volume overlap and random forest misclassification indices for *species X* in *species Y*, and the pairwise transformed PC volume overlap and random forest misclassification indices for *species Y* in *species X* in our analysis.

### Acoustic feature distances and phylogenetic analyses

To identify the acoustic features of song that correlate with evolutionary relatedness, we tested whether species distances along acoustic dimensions mapped on to the species phylogeny. To test for broad cladistic groupings of species by phylogeny, we used the Fowlkes-Mallows Index (FMI)^118^ for comparing the hierarchical clustering (topology) in the phylogeny to that of the acoustic distances. In order to acquire the hierarchical clustering in acoustic features, we first calculated the pairwise acoustic distances between species for each of our acoustic features (i.e., PCs 1–3 as well as our 21 acoustic features). The distributions of acoustic feature values were z-scored and binned spanning the minimum and maximum numbers of the data in increments of 0.01 in order to standardize the range and frequencies across features and species: 2501 bins for raw features and 1901 bins for PCs. We then calculated the pairwise distances between each species for each acoustic feature by calculating the sum of squared differences (hereafter “acoustic distances”) between the histograms, and used these values to produce the distance matrices for each acoustic feature. Acoustic distances were normalized to range between 0 (no distance) and 1 (most distance) and used to construct dendrograms using the *hclust* function for hierarchical clustering in R^99^. We used the *FM_index* function in the dendextend package^119^ for R for FMI cluster analysis. We used *k* = *3* to test whether species will group into their biogeographical clades (i.e., Australian, African, and Southeast Asian clusters) when clustered by acoustic distances. FMI tests the null hypothesis that the acoustic feature dendrograms are random reshufflings of the phylogenetic tree and provides an index for similarity (0 [dissimilar] to 1 [similar]). We used this index as well as the provided mean and variance for the expectancy value in the *pnorm* function of R in order to calculate the one-tailed p-value under the null hypothesis^118,119^ (i.e., trees are dissimilar; rejected at *α* = *0.05* when the observed FMI is significantly greater than the critical value).

In addition to biogeographical phylogenetic groupings, we wanted to test if the acoustic components that explained the greatest amount of variance in syllables (i.e., PCs 1–3) suggest phylogenetic constraint (i.e., predicted by relatedness under models of evolution)^16,77,120^ over lability (e.g., due to selection pressures not accounted for by the selected model of evolution)^6,70^. Testing for phylogenetic signal allows us to detect patterns in acoustic feature distributions that correlate with phylogenetic relatedness^47,121^. We produced a pruned phylogenetic tree for the species (see Figs. 1, 6a) extracted from a multi-locus estrildid phylogeny^48^. We used the acoustic distance matrices and the pruned phylogenetic tree in the *EMMantel* function (Brownian model; 1000 permutations) provided by^122^. By incorporating an evolutionary model to the Mantel test (EM-Mantel), we tested the probability of obtaining the observed pairwise acoustic distances if these features evolved under the specified model of evolution (i.e., Brownian motion)^122^. The Brownian motion EM-Mantel procedure tests the hypothesis that the observed species acoustic feature relationships are greater than or equal to (i.e., one-tailed) those expected to arise as a simple function of the evolutionary time between species.

Phylogenetic signal testing via the Mantel test is a two-step procedure^122^. First, we conducted the standard Mantel test on the phylogenetic distance and the acoustic distance matrices of species. If the standard Mantel test detected a significant correlation between phylogenetic distance and acoustic distance, we ran the EM-Mantel test for phylogenetic signal detection on those features. We applied false discovery rate (FDR) correction on all tests involving multiple features. We first conducted the two-step EM-Mantel procedure on the acoustic feature PCs. We then conducted the two-step procedure on all raw acoustic features that significantly loaded onto the PCs that had statistically significant EM-Mantel results.

To explore possible morphological constraints on vocal production in these species, Mantel tests with phylogenetic permutations (ppMantel) were performed to test the relationship between frequency feature and body size when controlling for phylogeny. The ppMantel performs comparative analyses of traits that are expressed as pairwise distances comparable to the independent contrasts for correlational analyses using raw trait data of individual species^123–125^. Tarsus length and body mass were used as morphometric measures of body size following previous conventions for correlating bird acoustics to body size^16,84,85^. Morphometric measures for the six wild type species were collected from the AVONET dataset^126^. Precise measures for the BF were sourced from Soma et al. to avoid confounds from discrepancies in morphometrics between this domesticated strain and its wild type^98^. We used the distance matrices for PC1 and each of our two morphometric features along with the pruned phylogenetic tree in the *PhyloMantel* function (1000 permutations) provided in the EvolQG package^127^ for R. Importantly, phylogenetic permutations account for the relationships between species (i.e., closely related species are more likely to be reassigned with each other, while species isolated in the phylogenetic tree will have higher self-permutations)^124^. In order to appropriately account for the nonrandom relatedness between our species, we set the parameter for phylogeny influence in the *PhyloMantel* function to its strongest (i.e., lowest) value (*k* = 1), which represents the furthest model from equal likelihood permutation (i.e., *k* = ∞; but see^124,128^ for effects of weakening phylogenetic control to assess different evolutionary models).

To quantify the contribution of phylogeny to acoustic differences between species, we calculated the phylogenetic inertia for each acoustic feature, including our 3 PCs^129^. The Mantel test statistic has previously been used for estimation of phylogenetic inertia, which is achieved by calculating the coefficient of determination (i.e., squared Mantel correlation coefficient)^129–132^. We squared the Mantel correlation coefficient reported by the EM-Mantel test in order to calculate phylogenetic inertia for each acoustic feature (see Supplementary Table S1). We plotted the rank-ordered phylogenetic inertia values in order to present features from highest to lowest inertia (i.e., from lowest to highest plasticity; Supplementary Fig. S5). Finally, we adopted a procedure for comparing the relative contributions of phylogenetic inertia and morphological differences to niche traits by comparing the effect sizes (i.e., correlation coefficients) from the EM-Mantel and ppMantel tests^83^. Specifically, we compared the EM-Mantel statistic for PC1 to the ppMantel statistics for PC1 and both body mass and tarsus length.

## Supplementary Information

Supplementary Information.

## Data Availability

The analysis code and datasets used here are available from the corresponding author on request.
