# Publisher Correction: Machine learning and statistical classification of birdsong link vocal acoustic features with phylogeny

**DOI:** 10.1038/s41598-023-35972-1

**Published:** 2023-05-31

**Authors:** Moises Rivera, Jacob A. Edwards, Mark E. Hauber, Sarah M. N. Woolley

**Affiliations:** 1 Department of Psychology, Hunter College and the Graduate Center, City University of New York, New York, NY 10065 USA; 2 Mortimer B. Zuckerman Mind, Brain, and Behavior Institute, Columbia University, New York, NY 10027 USA; 3 Department of Psychology, Columbia University, New York, NY 10027 USA; 4 Department of Evolution, Ecology, and Behavior, School of Biological Sciences, University of Illinois at Urbana-Champaign, Urbana, IL 61801 USA; 5 Zuckerman Institute at Columbia University, Jerome L. Greene Science Center, 3227 Broadway, L3.028, New York, NY 10027 USA

Correction to: *Scientific Reports* 10.1038/s41598-023-33825-5, published online 01 May 2023

The Supplementary Information file published with this Article contained a text error in the Figure S1 legend and a stray object in Figure S4. That Supplementary Information file is provided below.

The errors have now been corrected in the Supplementary Information file that accompanies the original Article.

## Supplementary Information

Supplementary Information.

