# Supplementary material for: Machine learning and statistical classification of birdsong link vocal acoustic features with phylogeny
Source: Sci Rep. 2023 May 1;13:7076. doi: 10.1038/s41598-023-33825-5 (PMC10151348; doi:10.1038/s41598-023-33825-5)
Supplement: Supplementary file 1 — Supplementary Information. [file 41598_2023_33825_MOESM1_ESM.pdf]

Machine learning and statistical classification of birdsong link vocal acoustic features with phylogeny

Moises Rivera, Jacob A. Edwards, Mark E. Hauber, and Sarah M. N. Woolley

Supplementary Table S1. Features, descriptions (based on warbleR documentation; see Araya-Salas & Smith-Vidaurre, 2017), PC loadings, feature importance scores, Fowlkes-Mallows indices (*FMI*), EM-Mantel correlation coefficients (*r*), and phylogenetic inertia estimates (R^2) for each of the 21 acoustic features measured from songs of the seven species. Superscripts denote features as components of frequency ([1]), power distribution ([2]), or spectrotemporal ([3]) features. Significant PC loadings (|Loading| > 0.218) and feature importance scores (i.e., first six rank-ordered scores) appear in bold. Significant phylogenetic measures following false discovery rate p-value correction for multiple testing denoted by asterisks at corresponding alpha levels (* < 0.05, ** < 0.01, *** < 0.001).

Acoustic Feature	Description	PC Loadings			Import.	Phylogen.
		PC1	PC2	PC3	Score	Measures
Duration[3]	Duration (ms) of syllable.	0.05	-0.08	**0.41**	**5.12**	*FMI* = 0.31<br>r = 0.13<br>R^2 = 0.018
Mean Frequency[1]	Weighted average (by amplitude) of the syllable frequency power spectrum (FPS; Hz).	**0.30**	-0.14	0.02	**8.49**	*FMI = 0.31*<br>*r* = 0.26<br>R^2 = 0.066
Standard Deviation of Mean Frequency[1,2]	Standard deviation of the weighted mean frequency (Hz).	**-0.22**	**-0.23**	-0.12	2.77	*FMI = 0.37*<br>*r* = 0.50<br>R^2 = 0.25
Peak Mean Frequency[1]	Frequency (Hz) from the mean frequency spectrum with the highest amplitude.	**0.30**	-0.12	-0.05	1.49	*FMI = 0.39*<br>*r* = 0.42<br>R^2 = 0.18
Minimum Fundamental Frequency[1]	Minimum frequency (Hz) of the first harmonic band in the syllable.	**0.31**	0.06	-0.10	**14.25**	*FMI = 0.62*<br>*r* = 0.34<br>R^2 = 0.12
Maximum Fundamental Frequency[1]	Maximum frequency (Hz) of the first harmonic band in the syllable.	**0.30**	-0.02	-0.05	**7.44**	*FMI = 0.39*<br>*r* = 0.55<br>R^2 = 0.30
Mean Fundamental Frequency[1]	Average frequency (Hz) of the first harmonic band in the syllable.	**0.31**	0.02	-0.08	**5.33**	*FMI = 0.39*<br>*r* = 0.60<br>R^2 = 0.36
Minimum Dominant Frequency[1]	Minimum of dominant frequencies (Hz) across the syllable. Dominant frequency is defined as the frequency with peak amplitude in the FPS at each sampling of the syllable along the time wave.	**0.30**	0.06	0.01	1.78	*FMI = 0.62*<br>*r* = 0.80<br>R^2 = 0.64
Maximum Dominant Frequency[1,2]	Maximum of dominant frequencies (Hz) across the syllable.	**0.28**	**-0.23**	0.02	2.19	*FMI = 0.37*<br>*r* = 0.01<br>R^2 = 0.00015

Acoustic Feature	Description	PC Loadings			Import.	Phylogen.
		PC1	PC2	PC3	Score	Measures
Mean Dominant Frequency[1]	Average of dominant frequencies (Hz) across the syllable.	**0.32**	-0.09	0.01	1.67	*FMI = 0.37*<br>r = 0.45<br>R^2 = 0.20
Dominant Frequency Range[2]	Range of dominant frequencies (Hz) across the syllable (MaxDom − MinDom).	0.04	**-0.34**	0.01	1.61	*FMI = 0.15*<br>r = - 0.36<br>R^2 = 0.13
Starting Dominant Frequency[1]	Dominant frequency (Hz) at the beginning of the syllable.	**0.28**	-0.10	-0.18	2.52	*FMI = 0.73**<br>r = 0.75<br>R^2 = 0.56
Ending Dominant Frequency[1]	Dominant frequency (Hz) at the end of the syllable.	**0.29**	0.00	0.11	1.37	*FMI = 0.31*<br>r = 0.51<br>R^2 = 0.26
Dominant Frequency Slope[3]	Change in dominant frequency across the syllable (Hz/ms). (EndDom − StartDom)/Duration.	0.01	0.11	**0.39**	3.30	*FMI = 0.17*<br>r = - 0.26<br>R^2 = 0.068
Skewness[2]	Asymmetry of the FPS. FPS may be left skewed (S < 0), right skewed (S > 0), or symmetrical (S = 0).	0.01	**0.38**	0.14	1.45	*FMI = 0.46*<br>r = - 0.19<br>R^2 = 0.036
Kurtosis[2]	Peakedness of the FPS. FPS may be platikurtic (K < 3), leptokurtic (K > 3), or normal (K = 3).	0.00	**0.33**	0.14	0.47	*FMI = 0.26*<br>r = - 0.26<br>R^2 = 0.068
Modulation Index[3]	Sum of absolute differences between adjacent dominant frequencies divided by the dominant frequency range. A modulation index of 1 means syllable is not modulated.	0.02	-0.21	**0.37**	3.55	*FMI = 0.26*<br>r = - 0.19<br>R^2 = 0.038
Spectral Entropy[2]	A measure of the energy distribution across frequencies. Noisy syllables (those whose energy is not "neatly" confound to frequency bands) will tend toward 1. Tonal syllables (those whose energy is confound to frequency bands) will tend toward 0. Described as "Wiener Entropy" in Tchernichovski et al. (2000).	-0.12	**-0.41**	-0.06	1.76	*FMI = 0.46*<br>r = 0.11<br>R^2 = 0.011

Acoustic Feature	Description	PC Loadings PC1	PC2	PC3	Import. Score	Phylogen. Measures
Temporal Entropy[3]	A measure of the energy distribution across time. Noisy syllables (those with sustained amplitudes or high spectral continuity [Tchernichovski et al., 2000]) will tend toward 1. Quiet syllables (those with low spectral continuity) will tend toward 0.	0.04	-0.07	**0.55**	2.09	*FMI = 0.37*<br>r = 0.16<br>R^2 = 0.026
Entropy[2,3]	Measure of the two-dimensional spectrotemporal energy distribution of the syllable (Spectral Entropy · Temporal Entropy). Noisy syllables will tend toward 1. Tonal syllables will tend toward 0.	-0.08	**-0.37**	**0.31**	1.20	*FMI = .67*<br>r = .12<br>R^2 = 0.014
Spectral Flatness[2]	Measure of how closely the FPS energy distribution resembles a uniform distribution (i.e., white noise). Similar to SpectEnt. Noisy syllables will tend toward 1. Tonal syllables will tend toward 0.	-0.15	**-0.32**	-0.17	**10.57**	*FMI = 0.46*<br>r = 0.01<br>R^2 = 0.00017

Supplementary Figure S1. Violin plots for species distributions along the 3 acoustic feature components. Horizontal line across each violin shows the distribution's median value. (a) Frequency features clustered species according to their biogeographic clades: Australian species (ZF, LF, DF) have low frequency values; African species (RF, CB, GW) have high frequency values range, and the Southeast Asian species (BF) spans the range between both clades. (b) Power distribution features do not cluster species according to biogeographic clades, on average: ZFs have the lowest values, consistent with the abundance of noisy (high entropy/spectral flatness), broadband harmonic syllables in their songs; LFs have the highest values, consistent with the abundance of low-harmonicity and low-noise (tonal) syllables in their song; all other species showed intermediate values (near PC2 = 0). (c) Spectrotemporal features do not cluster species according to biogeographic clades, on average. BFs had the lowest values, consistent with the abundance of very short durations and down-sweeping syllables in their songs. All other species showed similar values to each other.

a.

b.

c.

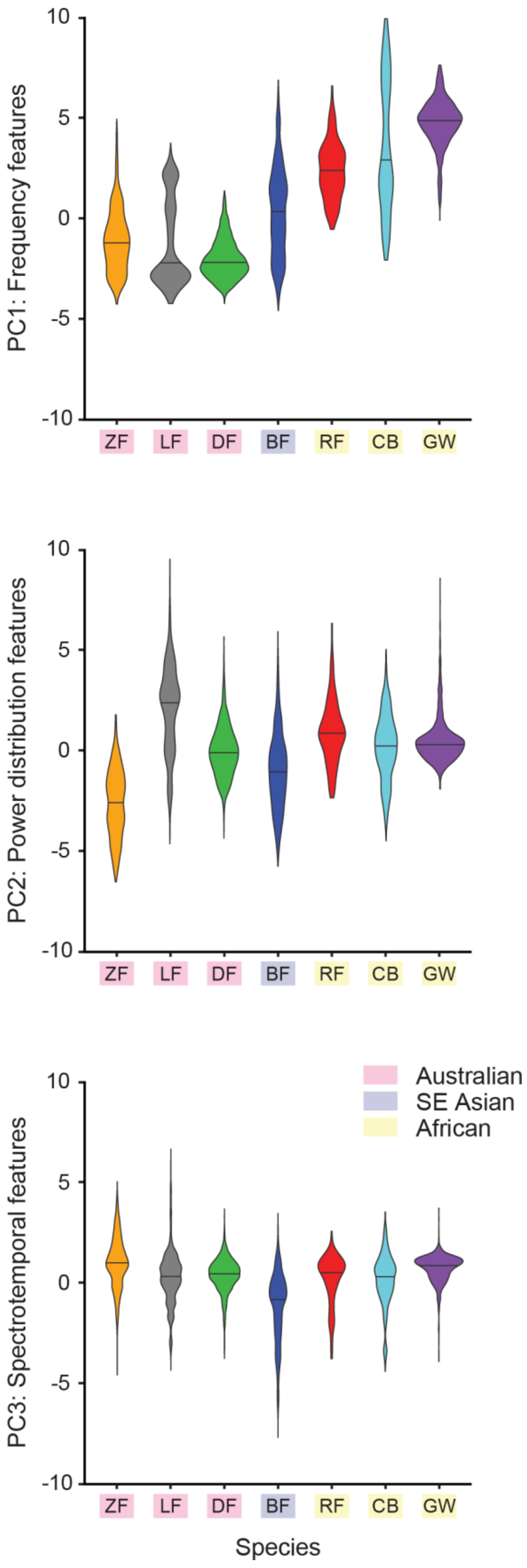

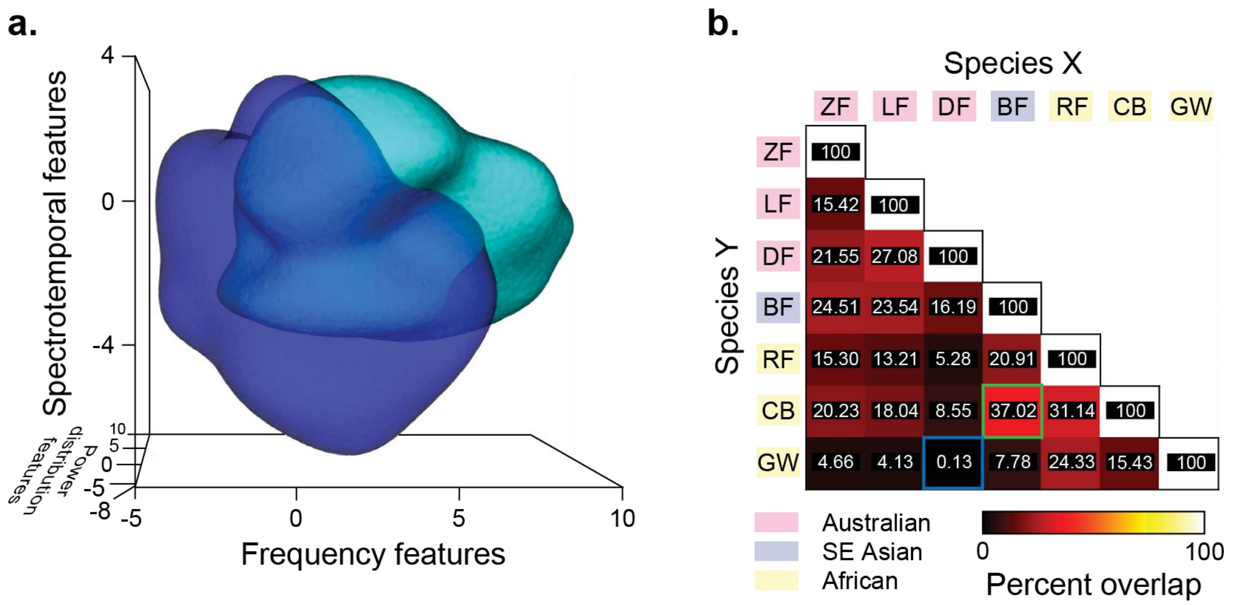

Supplementary Figure S2. Overlap of volumes in PC space differed in pairwise species comparisons. (a) The feature volumes of BF (blue) and CB (cyan) syllables show high overlap volume (> 37%). (b) Pair-wise Jaccard similarity indices between species. Jaccard similarity index provides a percentage of the total volume occupied by the two species that is shared between them. The highest overlap volume is between BF and CB (green square), while the lowest is between DF and GW (0.13%; blue square). High overlap between BF and CB is likely due to the moderate-to-high frequency harmonics and variable entropy in syllables – characteristics shared by both species. CB syllables tend to be higher in frequencies and have more upsweeps than BF syllables. Low overlap between DF and GW is due to the dissimilar syllable acoustics of these songs. DF syllables are harmonic with relatively low fundamental frequencies and slower spectrotemporal modulations, while GW syllables are tonal, with high fundamental frequencies and faster spectrotemporal modulations.

Supplementary Figure S3. Species distributions for syllable (a) minimum, (b) maximum, and (c) mean fundamental frequencies. Horizontal line across each violin shows the distribution's median value. Overall, Australian species (ZF, LF, DF) have lower fundamental frequencies than African species (RF, CB, GW), and BF spans the range between these two clades.

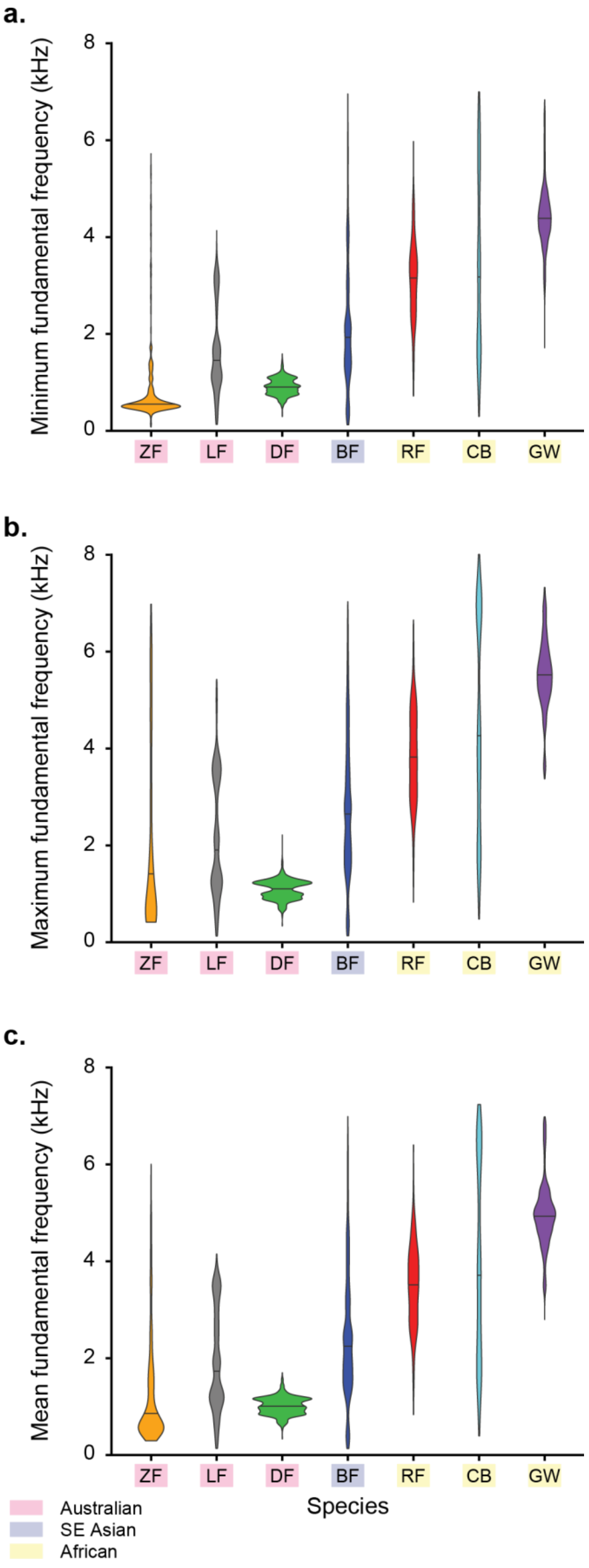

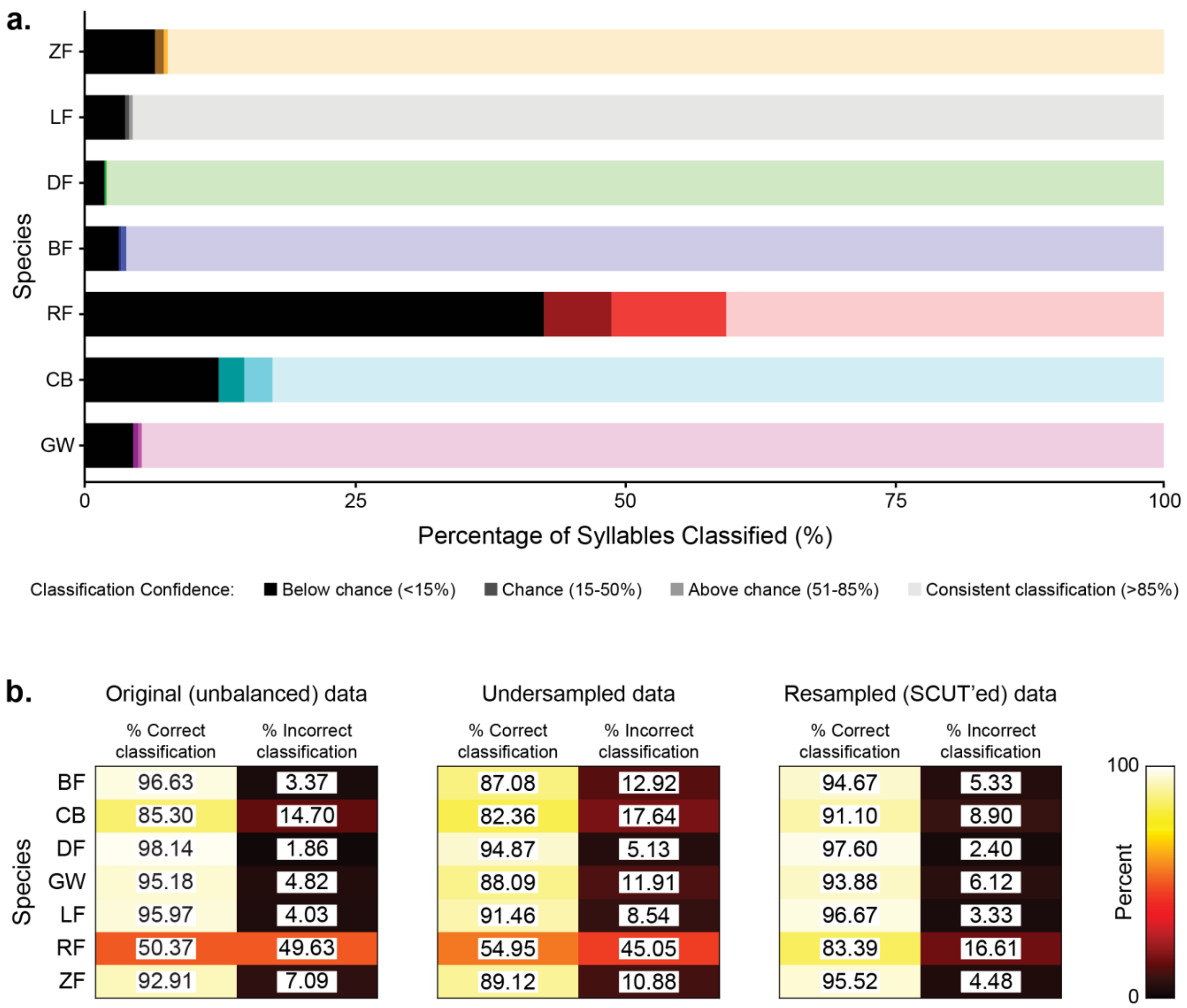

Supplementary Figure S4. Random forest model performance was robust across iterations and with unbalanced sample sizes. (a) Classification confidence for species syllables across 100 iterations of random forest in the 6-feature model. The percent of random forests that correctly classified each syllable across the 100 iterations (shading gradients in legend) represents the confidence (i.e., consistency) with which syllables are correctly labelled. We calculated the percentage of syllables (x-axis) that were classified at four levels of confidence within each species: Below Chance (<15%), Chance (15-50%), Above Chance (51-85%), Consistent Classification (>85%). 15% represents the minimum majority needed to assign a species label assuming uniform probabilities (1/(no. species) = 1/7 = 14.29 < 15). 51% represents the minimum majority needed to assign a species label assuming dichotomous probability (i.e., 50% chance it will be correctly labelled, 50% chance it will not). 86% (i.e., >85%) represents the minimum majority needed to accept syllable labelling beyond significant consideration for any other single species (i.e., a syllable correctly labelled as belonging to *species X* 86% of the time could at most only be labelled as belonging to *species Y* 14% of the time, which is below our 15% threshold for chance). Overall, there was high consistency in the classification of syllables across random forest iterations for all species, with RF classifications being the least consistent, followed by CB. (b)

Percent correct and incorrect classifications for each species in the 6-feature model using the original (left), under-sampled (centered), and resampled (right) data sets. Classification accuracy using the under-sampled data set decreased for all species except RFs, which showed an increase from 50% to 55%. Classification accuracy using the resampled dataset showed changes in species consistent with their resampling: over-sampled species (CB, RF, ZF) increased in classifications, while under-sampled species (BF, DF) decreased in classification. Changes in LF (0.7% increase) and GW (1.3% decrease) classifications were inconsistent with their resampling but are minor compared to considerable increases in classifications of CB (6% increase) and RF (33% increase) syllables. Overall, our results suggest that although the random forest model is robust against unbalanced sample sizes among species, species-wise classifications could be improved with greater sample sizes.

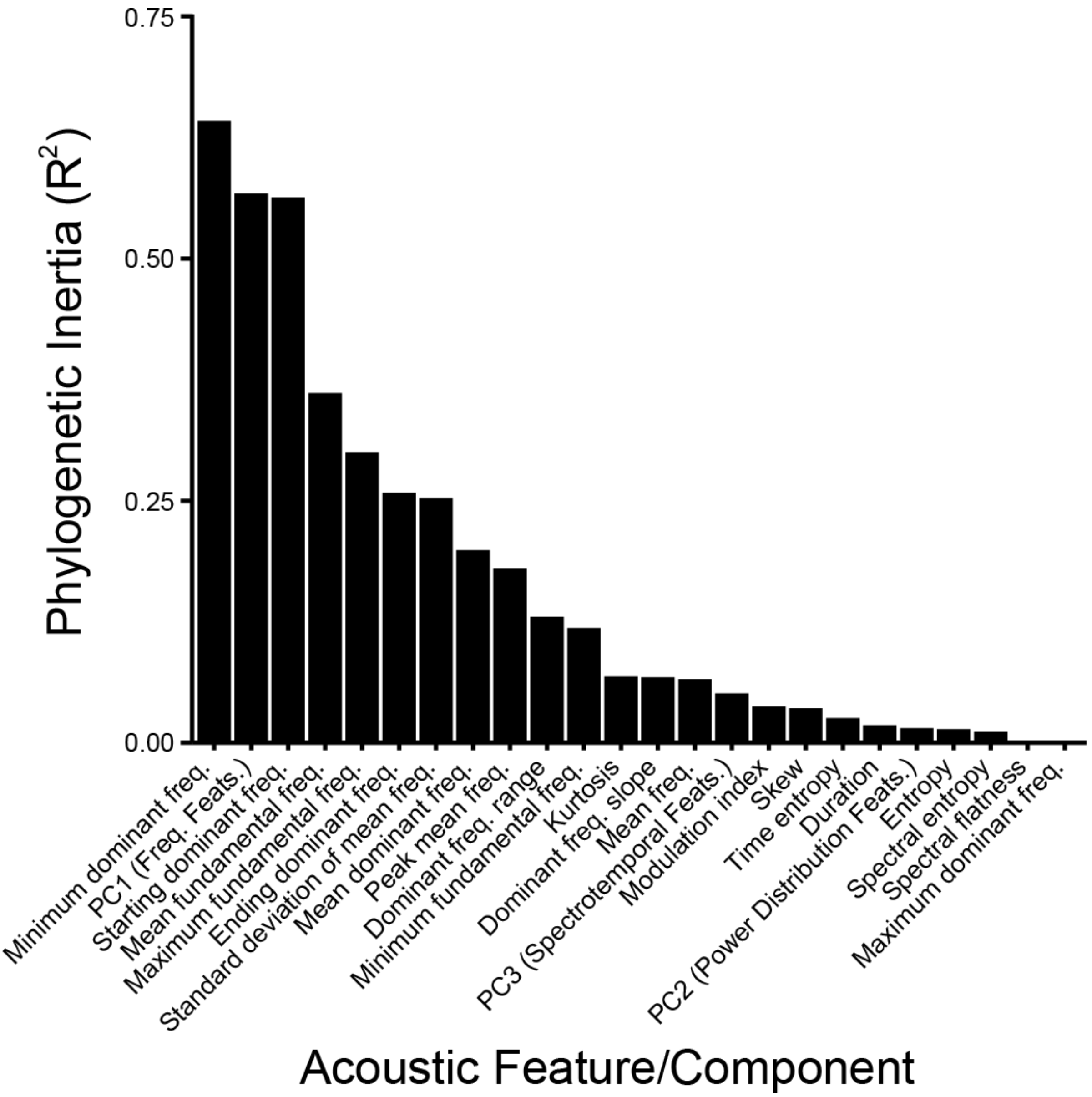

Supplementary Figure S5. Rank-ordered phylogenetic inertia estimates for each of our acoustic features and components. The inertia estimate is obtained by calculating the coefficient of determination from the EM-Mantel test for each feature (i.e., the Mantel correlation statistic squared, R^2). Acoustic features are plotted in order of decreasing phylogenetic inertia values, such that features toward the left of the plot have more influence of phylogeny in their evolutionary change (i.e., greater phylogenetic inertia), while features toward the right of the plot have less influence of phylogeny in their evolutionary change (i.e., lower phylogenetic inertia and greater plasticity for adaptation). Generally, we find that frequency features (including PC1) experience moderate-to-high phylogenetic constraint (e.g., phylogenetic inertia for PC1 = 57%), while power distribution and spectrotemporal features (including PCs 2 & 3) experience less phylogenetic constraint (i.e., phylogenetic inertia for PC2 = 2%, PC3 = 5%), suggesting these latter features may be suitable candidates for adaptation and the plasticity needed to avoid interspecific acoustic competition.
